# Vitamin C enhances anticancer activity in methotrexate-treated Hep3B hepatocellular carcinoma cells

**DOI:** 10.3892/or.2014.3289

**Published:** 2014-06-25

**Authors:** GIOU-TENG YIANG, PEI-LUN CHOU, YU-TING HUNG, JEN-NI CHEN, WEI-JUNG CHANG, YUNG-LUEN YU, CHYOU-WEI WEI

**Affiliations:** 1 Department of Emergency Medicine, Taipei Tzu Chi Hospital, Buddhist Tzu Chi Medical Foundation, New Taipei 231, Taiwan, R.O.C.; 2 Department of Emergency Medicine, School of Medicine, Tzu Chi University, Hualien 970, Taiwan, R.O.C.; 3 Division of Allergy-Immunology-Rheumatology, Department of Internal Medicine, Saint Mary’s Hospital Luodong, Yilan 265, Taiwan, R.O.C.; 4 Department of Internal Medicine, School of Medicine, College of Medicine, Taipei Medical University, Taipei 110, Taiwan, R.O.C.; 5 Department of Nutrition, Master Program of Biomedical Nutrition, Hungkuang University, Shalu, Taichung 433, Taiwan, R.O.C.; 6 Department of Food Science and Biotechnology, National Chung Hsing University, Taichung 402, Taiwan, R.O.C.; 7 Graduate Institute of Cancer Biology, and Center for Molecular Medicine, China Medical University, Taichung 404, Taiwan, R.O.C.; 8 Department of Biotechnology, Asia University, Taichung 413, Taiwan, R.O.C.

**Keywords:** methotrexate, vitamin C, apoptosis, hepatocellular carcinoma

## Abstract

Methotrexate (MTX) has been widely used for rheumatoid arthritis therapy for a long time. MTX is also used as an anticancer drug for various tumors. However, many studies have shown that high-dose MTX treatment for cancer therapy may cause liver and renal damage. Alhough the mechanisms involved in MTX-induced liver and renal damage require further research, many studies have indicated that MTX-induced cytotoxicity is associated with increases in oxidative stress and caspase activation. In order to reduce MTX-induced side-effects and increase anticancer efficiency, currently, combination treatments of low-dose MTX and other anticancer drugs are considered and applied for various tumor treatments. The present study showed that MTX induces increases in H_2_O_2_ levels and caspase-9/-3 activation leading to cell death in hepatocellular carcinoma Hep3B cells. Importantly, this study is the first to demonstrate that vitamin C can efficiently aid low-dose MTX in inducing cell death in Hep3B cells. Therefore, the present study provides a possible powerful therapeutic method for tumors using a combined treatment of vitamin C and low-dose MTX.

## Introduction

Methotrexate (MTX) is one of the most popular and safe antirheumatic drugs under the applied treatment dose (1,2). In order to obtain a better curative effect in clinical cases, MTX is also used in combination with other drugs for rheumatoid arthritis treatment (1,3,4). In addition, MTX is also used as an anticancer drug (5). Recently, MTX has been widely applied for the treatment of various cancers, such as hepatoma, osteosarcoma, leukemia, lymphoma, gastric, breast, head and neck cancers (5–9). Many studies have demonstrated that MTX induces cancer cell death via apoptotic death pathways (10–14). Apoptotic death pathways can be divided into caspase-dependent and caspase-independent cascades (15,16). Concerning the MTX-induced apoptotic pathways, most studies have shown that MTX induces apoptosis via caspase-dependent cascades in many cancer cell lines (17–21). However, some studies have indicated that MTX can induce apoptosis via caspase-independent cascades in osteosarcoma cells (22,23). The present study found that MTX-induced apoptosis in Hep3B cells is via the caspase-dependent cascade, similar to most other studies (17–21).

Two major caspase cascade pathways have been reported (24–26). One is the caspase-8/-3 cascade, known as the extrinsic death receptor pathway (CD95/APO-1/Fas receptor) (27–29). Another is the caspase-9/-3 cascade, known as the intrinsic mitochondrial death pathway (27,30,31). Some studies have shown that MTX-induced apoptosis is mediated by the caspase-9/-3 cascade pathway in choriocarcinoma, breast cancer, oral squamous carcinoma and hepatoma cells (18,19,21,32,33). In contrast, some studies demonstrated that MTX-induced apoptosis is mediated through the caspase-8/-3 cascade pathway in breast cancer, hepatoma and leukemia cells (17,33,34). The present study showed that MTX activates the caspase-9/-3 cascade in Hep3B cells, but not the caspase-8/-3 cascade.

Previously, many studies have shown that high-dose MTX treatment can induce increased oxidative stress, resulting in renal and liver damage (3,35–37). However, the specific reactive oxygen species (ROS) induced by MTX treatment have not been identified. O_2_^−^ and H_2_O_2_ are ROS families generally existing in many cells. By using the lucigenin-amplified method (38–40), our results are the first to demonstrate that MTX can induce increases in H_2_O_2_ levels, but not O_2_^−^ levels.

Considering that high-dose MTX treatments can cause renal and liver damage (35–37), combination treatments of low-dose MTX and other anticancer drugs are suggested and applied during clinical cancer therapy in order to enhance the anticancer effects and decrease MTX-induced side-effects (9,10,12,18,41). However, not all anticancer agents can enhance the anticancer effects of low-dose MTX. A recent study showed that aspirin can antagonize the MTX-induced cytotoxic effect on lung cancer cells (42). Alternatively, there have been many reports on the antioxidant activities of vitamin C (43–47). Moreover, some studies have demonstrated that vitamin C can exert anticancer activities in various cancer cells (48–52). The present study demonstrated that vitamin C can diminish MTX-induced increases in H_2_O_2_ levels. On the other hand, it is worth noting that vitamin C can help low-dose MTX exert a cytotoxic effect on Hep3B cells. Taken together, the study demonstrated that MTX activates the caspase-9/-3 cascade and induces increased H_2_O_2_ levels, causing cell cytotoxicity in Hep3B cells, while more importantly, the present study is the first to demonstrate that vitamin C enhances the anticancer efficiency in MTX-treated Hep3 cells.

## Materials and methods

### Chemicals and materials

Methotrexate was purchased from Pfizer Inc. MTT assay kit was purchased from Bio Basic Canada Inc. Hoechst 33342, vitamin C, lucigenin and luminol were purchased from Sigma. Caspase-3 like substrate (Ac-DEVD-pNA), caspase-8 substrate (Ac-IETD-pNA) and caspase-9 substrate (Ac-LEHD-pNA) were purchased from AnaSpec, Inc. (San Jose, CA, USA). Fetal bovine serum (FBS), Dulbecco’s modified Eagle’s medium (DMEM), non-essential amino acid, L-glutamine and penicillin/streptomycin were purchased from Gibco-BRL.

### Cell cultures

Hep3B cells were cultured in DMEM containing 10% FBS, 2 mM L-glutamine, 100 IU/ml penicillin/streptomycin, and 0.1 mM non-essential amino acids. The cells were cultured at 37°C in a humidified atmosphere containing 5% CO_2_.

### Cell viability assay

Hep3B cell viability was assessed using the MTT assay method according to the manufacturer’s instructions. In brief, Hep3B cells were maintained in each well of 96-well culture plates. Every 24 h, the control group and experimental groups were subjected to the MTT assay kit. After 3 h of incubation, absorbance at 570 nm for each well containing Hep3B cells was detected under a multi-well ELISA reader (Molecular Devices). Cell viability was calculated using the following formula: A570 experimental group/A570 control group × 100%.

### Nuclear condensation and DNA fragmentation

Apoptotic cells were identified by nuclear condensation and DNA fragmentation using Hoechst 33342 staining. Cells were treated with 10 μg/ml Hoechst 33342 for 10 min. Nuclear condensation and DNA fragmentation were observed under a fluorescence microscope (excitation, 352 nm; emission, 450 nm) (53,54).

### Caspase activity assay

Caspase activity assays were executed according to previous studies (55,56). In brief, Hep3B cells were lysed with a lysis buffer (50 mM Tris-HCl, 120 mM NaCl, 1 mM EDTA, 1% NP-40, pH 7.5) and protease inhibitors. After centrifugation (15,000 × g, 30 min, 4°C) cell pellets were collected. The working solutions containing 40 μl cell lysates (80 μg total protein), 158 μl reaction buffer (20% glycerol, 0.5 mM EDTA, 5 mM dithiothreitol, 100 mM HEPES, pH 7.5) and 2 μl fluorogenic caspase substrate (Ac-LEHD-pNA, Ac-DEVD-pNA or Ac-IETD-pNA) were incubated at 37°C for 6 h. Fluorogenic substrate cleavage was determined at 405 nm in an ultra-microplate reader (BioTek Instruments). The fold increase in caspase activity was calculated using the following formula: (A405 experimental group - A405 control group)/A405 control group.

### Determination of H_2_O_2_ and O_2_^−^ levels

H_2_O_2_ and O_2_^−^ levels were examined by using lucigenin-amplified chemiluminescence according to the lucigenin-amplified method (57,58). In brief, for H_2_O_2_ levels, the sample (200 μl) was mixed with 0.2 mmol/l luminol solution (100 μl). After that, the mixture was measured with a chemiluminescence analyzing system (CLA-FSI; Tohoko Electronic Industrial Co., Ltd., Miyagi, Japan) for determination. For O_2_^−^ levels, 200 μl of the sample was mixed with 0.1 mmol/l of lucigenin solution (500 μl), and was then measured by the CLA-FSI chemiluminescence analyzing system.

### Statistical analysis

Experimental data were calculated from three independent triplicate experiments and are presented as the mean values of the chosen triplicate groups. These experimental data are shown as means with standard deviations.

## Results

### MTX exerts dose-dependent and time-dependent anticancer effects on Hep3B cells

**Figure 1:**
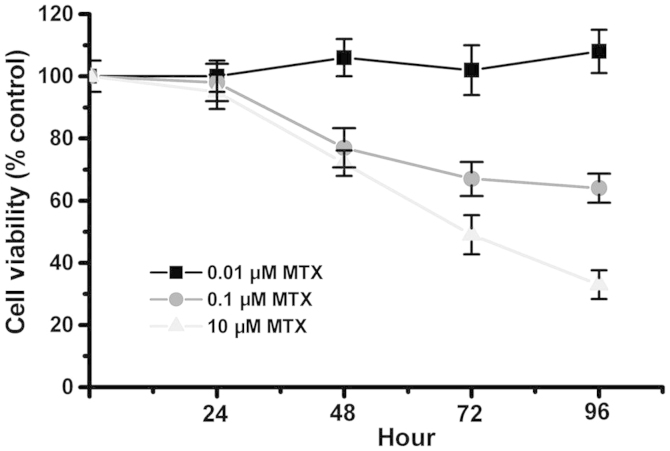
Cell viability. Hep3B cells were treated with 10, 0.1 and 0.01 μM MTX. Cell viability was measured with MTT assay every 24 h and presented as A570 experimental group/A570 control group × 100%. Data were collected from 4 independent experiments and are presented as mean ± SD.

In clinical cases, 10–25 mg/week MTX (~0.1 μM/day) is a safely applied dose for rheumatoid arthritis treatment (1,2,59). In the present study, 0.1 μM (treatment-dose), 0.01 μM (low-dose) and 10 μM (high-dose) MTX were used for studying the anticancer effects on Hep3B cells. Hep3B cell viability decreased in the 0.1 and 10 μM MTX treatment groups, but did not decrease in the 0.01 μM treatment group (Fig. 1). In addition, the 10 μM MTX treatment group showed a stronger cytotoxic effect in the Hep3B cells than the 0.1 μM MTX treatment group. These data suggest that MTX exerts a dose-dependent anticancer effect on Hep3B cells. In addition, cell viability was observed over different MTX incubation times, with results showing that the cell viability decreased incrementally in the 0.1 and 10 μM MTX groups. The present study indicates that MTX exerts a dose-dependent and time-dependent anticancer effect on Hep3B cells.

### MTX induces apoptosis and activates the caspase-9/-3 cascade in Hep3B cells

**Figure 2:**
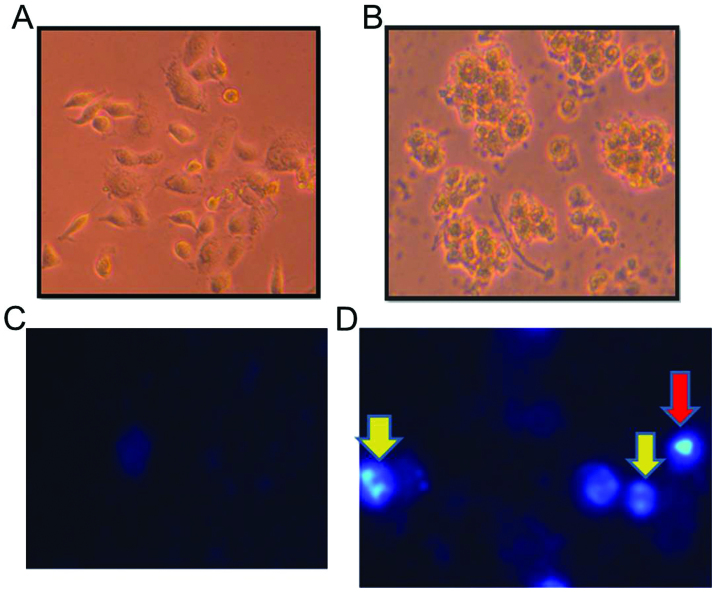
Cell morphology, nuclear condensation and DNA fragmentation. (A) Control cells, (B) MTX-treated cells. Hep3B cells were treated with or without 10 μM MTX for 96 h, and cell morphology was observed under a phase-contrast microscope. (C) Control cells, (D) MTX-treated cells. Cells were treated with or without 10 μM MTX for 96 h, and nuclear condensation and DNA fragmentation were observed using Hoechst 33342 staining. Note that nuclear condensation (red arrow) and DNA fragmentation (yellow arrow) were noted in the MTX-treated cells.

**Figure 3:**
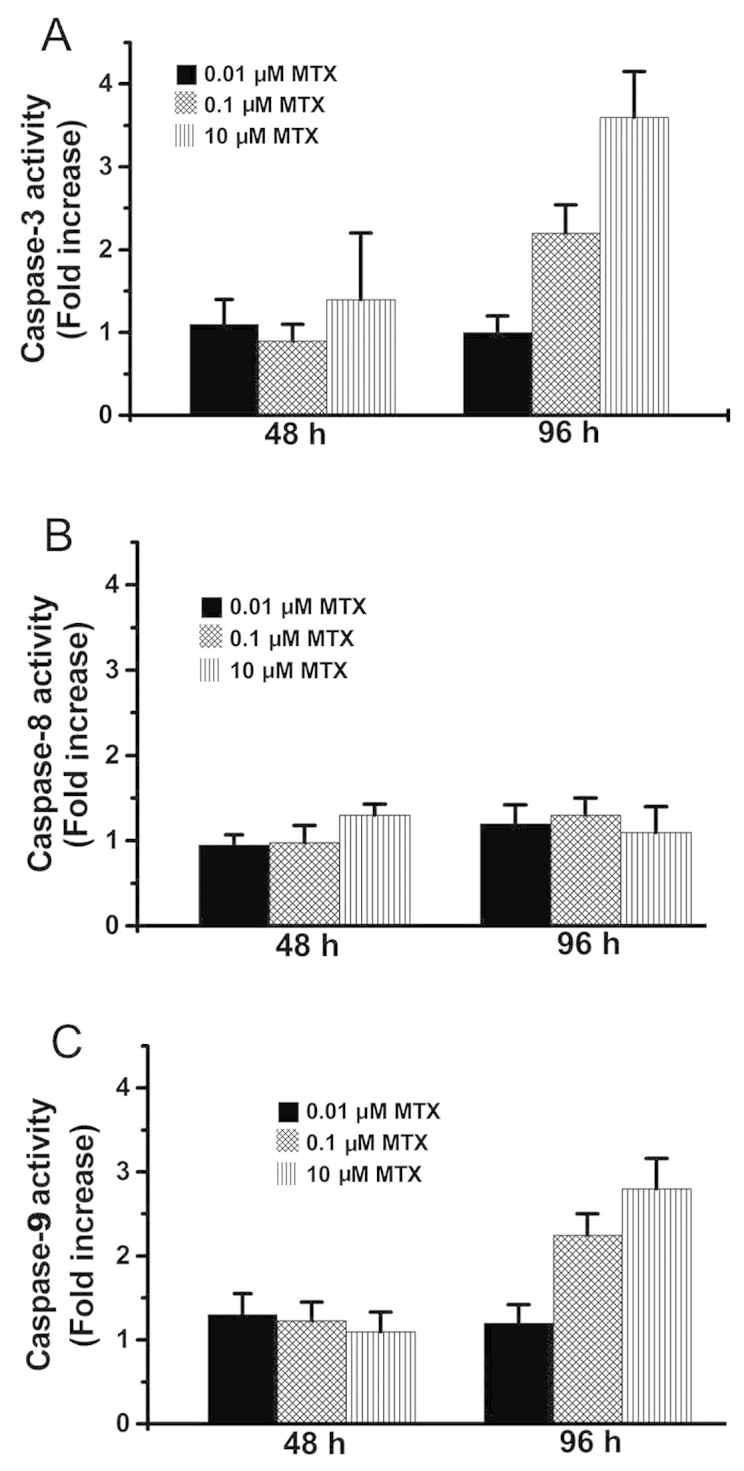
Caspase activity. (A) Caspase-3, (B) caspase-8 and (C) caspase-9 activities were determined at 48 and 96 h in Hep3B cells treated with 0.01 μM, 0.1 μM and 10 μM MTX. Note that caspase-3 and caspase-9 activities were significantly increased in the 0.1 and 10 μM MTX-treated cells. Data were obtained from 3 independent experiments and are presented as means ± SD.

The study investigated whether MTX induces apoptosis in Hep3B cells. Cell morphology was observed under a phase-contrast microscope. Hep3B cells survived with morphology intact in the control group (Fig. 2A). However, dead cells were noted in the MTX treatment group (Fig. 2B). In addition, nuclear condensation and DNA fragmentation are apoptotic features and can be observed using a nuclear staining method, as previously described (55,60). Compared with the control group (Fig. 2C), nuclear condensation and DNA fragmentation were noted in the MTX-treated group (Fig. 2D). The results indicate that MTX induced apoptosis in the Hep3B cells. Next, caspase activation was determined in the MTX-treated Hep3B cells by using a substrate cleavage assay (56,61). As shown in Fig. 3A, caspase-3 activity increased in the Hep3B cells at 96 h following treatment with 0.1 and 10 μM MTX while caspase-3 activity did not increase in Hep3B cells following treatment with 0.01 μM MTX. Caspase-9 activity also increased in the 0.1 and 10 μM MTX-treated Hep3B cells at 96 h but did not increase in the 0.01 μM MTX-treated cells (Fig. 3C). However, there was no obvious increase in caspase-8 activity among the MTX-treated Hep3B cells (Fig. 3B). These results suggest that MTX (10 and 0.1 μM) induced apoptosis in the Hep3B cells via the caspase-9/-3 cascade but not via the caspase-8/-3 cascade.

### MTX causes increases in H_2_O_2_ levels but not O_2_^−^ levels in Hep3B cells

**Figure 4:**
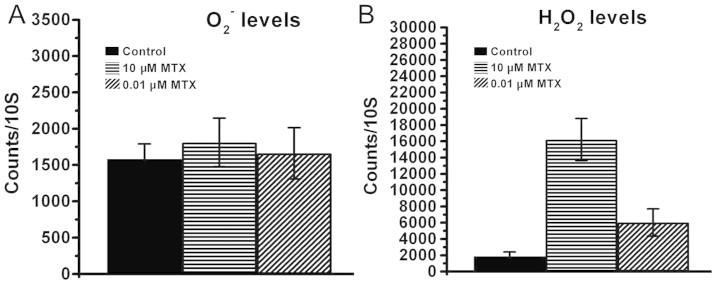
O_2_^−^ and H_2_O_2_ levels. (A) O_2_^−^ counts and (B) H_2_O_2_ counts are presented in the control cells, 0.01 μM MTX-treated cells, and 10 μM MTX-treated cells. O_2_^−^ and H_2_O_2_ levels were determined after treatment for 1 h using a lucigenin-amplified method. Data were collected from 4 independent experiments and are presented as the means ± SD.

Previous studies have shown that MTX can cause cell cytotoxicity associated with increases in reactive oxygen species (ROS) (35–37). Prior to the present study, the literature has not yet identified which ROS is induced by MTX treatment. Both O_2_^−^ and H_2_O_2_ belonging to ROS commonly exist in cells. Therefore, O_2_^−^ and H_2_O_2_ levels were examined according to the lucigenin-amplified method (57,58). The present study found that MTX did not raise O_2_^−^ levels in the Hep3B cells (Fig. 4A). However, both high-dose MTX and low-dose MTX raised H_2_O_2_ levels in the Hep3B cells (Fig. 4B). Therefore, the MTX-induced ROS increase is related to H_2_O_2_ levels but not related to O_2_^−^ levels in the Hep3B cells.

### Vitamin C reduces the increase in H_2_O_2_ levels and enhances the anticancer efficacy in MTX-treated Hep3B cells

**Figure 5:**
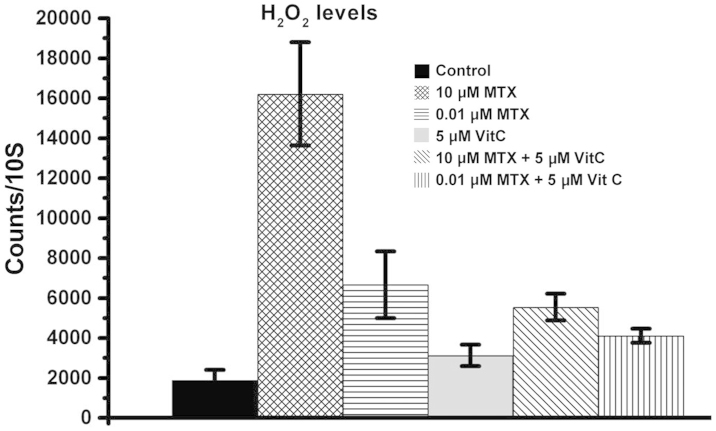
H_2_O_2_ levels. H_2_O_2_ counts are presented in control cells, MTX-treated cells, vitamin C-treated cells, and MTX plus vitamin C-treated cells. H_2_O_2_ levels were determined after treatment for 1 h using a lucigenin-amplified method. Data were collected from 4 independent experiments and are presented as means ± SD.

**Figure 6:**
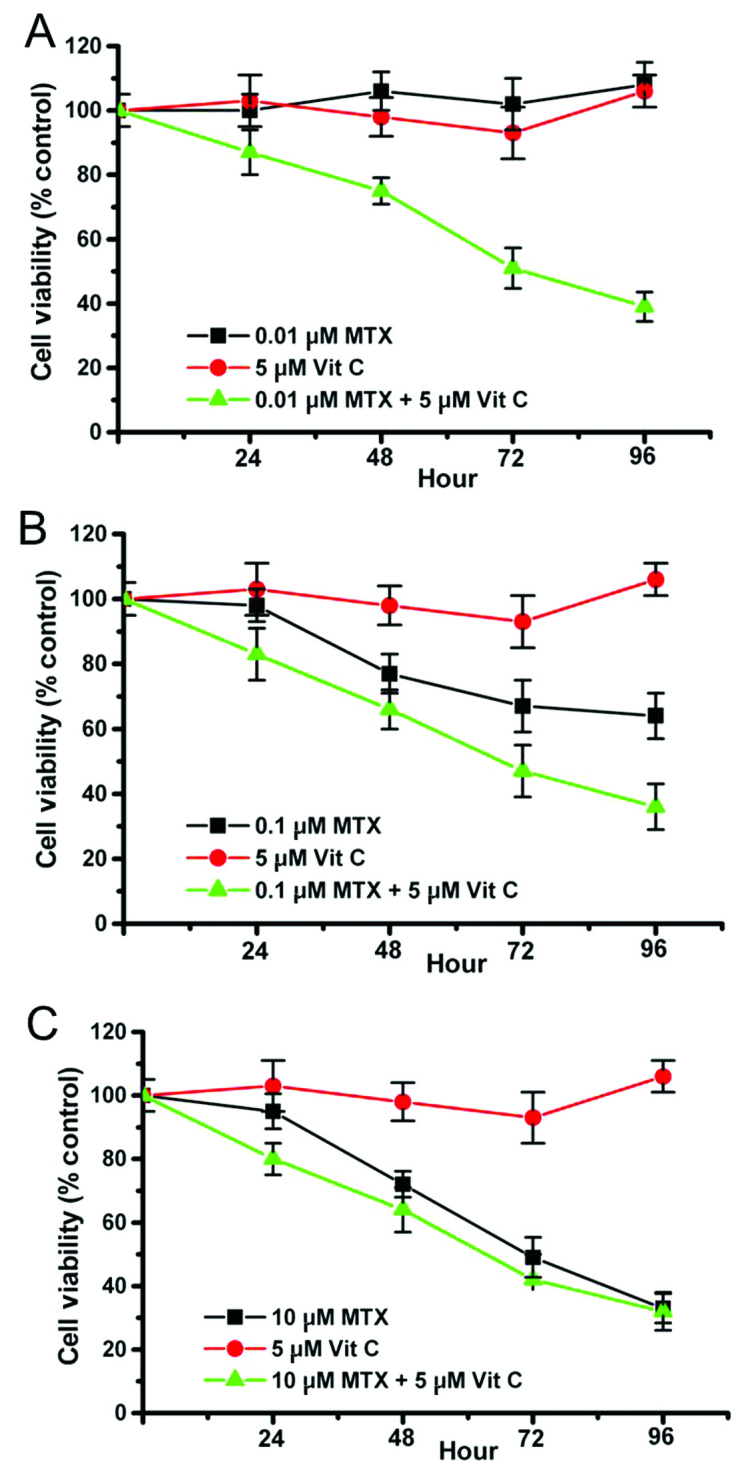
Cell viability. (A) Hep3B cells were treated with 0.01 μM MTX, 5 μM vitamin C, and a combination of 0.01 μM MTX and 5 μM vitamin C. (B) Hep3B cells were treated with 0.1 μM MTX, 5 μM vitamin C or a combination of 0.1 μM MTX and 5 μM vitamin C. (C) Hep3B cells were treated with 10 μM MTX, 5 μM vitamin C or a combination of 10 μM MTX and 5 μM vitamin C. Cell viability was measured with MTT assay every 24 h and is presented as A570 experimental group/A570 control group × 100%. Data were collected from four independent experiments and are presented as means ± SD.

Many studies have demonstrated that vitamin C can prevent oxidative stress-induced cell damage (43–47). Considering that MTX induces oxidative stress resulting in cell damage (35–37), this study examined whether vitamin C could decrease H_2_O_2_ levels, essentially inhibiting MTX-induced cytotoxicity in Hep3B cells. As shown in Fig. 5, the group receiving a combination treatment of vitamin C and 10 μM MTX had lower H_2_O_2_ levels than the 10 μM MTX group. Similarly, the vitamin C and 0.01 μM MTX combination treatment group had lower H_2_O_2_ levels than the 0.01 μM MTX group. These data indicate that vitamin C reduced the MTX-induced H_2_O_2_ levels. However, to our surprise, vitamin C did not attenuate cell cytotoxicity in the MTX-treated Hep3B cells. On the contrary, our data showed that vitamin C enhanced the anticancer efficacy in MTX-treated Hep3B cells (Fig. 6). As shown in Fig. 6A and B, combination treatments of 5 μM vitamin C and MTX (0.01 or 0.1 μM) exerted a stronger anticancer effect on Hep3B cells than MTX treatment alone. It is worth noting that 0.01 μM MTX alone or 5 μM vitamin C alone did not have a significant cytotoxic effect on Hep3B cells, whereas a combination treatment of 0.01 μM MTX and 5 μM vitamin C did induce a cytotoxic effect on Hep3B cells (Fig. 6A). While vitamin C did not enhance the 10 μM MTX-induced cytotoxic effect on Hep3B cells (Fig. 6C), the present study was important in indicating that vitamin C can assist low-dose MTX exert an anticancer effect on Hep3B cells.

## Discussion

Previous reports have revealed that MTX-induced cytotoxicity is related to increased reactive oxygen species (ROS) (35–37). However, no study has shown which ROS are induced following MTX treatment. In the present study, two types of ROS, O_2_^−^ and H_2_O_2_, were measured. H_2_O_2_ levels in MTX-treated cells rose significantly while O_2_^−^ levels did not. In addition, it is well known that glutathione can convert toxic H_2_O_2_ into non-toxic H_2_O. We suggest that the increase in H_2_O_2_ levels is a possible and important reason why N-acetyl cysteine (NAC), a clinical drug for glutathione synthesis, is used for MTX-induced cell damage (35,38,39,62). On the other hand, high-dose MTX-induced H_2_O_2_ level increases were higher than low-dose MTX-induced H_2_O_2_ level increases (Fig. 4B). Our data also showed that MTX induced cytotoxicity in a dose-dependent manner (Fig. 1). Taken together, we consider increases in the H_2_O_2_ level to be one factor resulting in the inhibition of cell survival following MTX treatment.

MTX has anticancer effects on various hepatoma cell lines, including HepG2, MHCC97, Huh7 and Morris 5123 cells (6,63–67). Alhough the mechanisms involved in the MTX-induced cytotoxic effects on different hepatoma cells remain undetermined, a previous study demonstrated that MTX-induced cytotoxic effects on HepG2 cells are related to the CD95 death receptor pathway (caspase-8/-3 cascade pathway), whereas MTX-induced cytotoxic effects on Huh7 and Hep3B cells are not related to death receptor pathways (65). Similarly, the caspase-8/-3 cascade pathway was also found not to be involved in MTX-treated Hep3B cells in the present study (Fig. 3B). This study further demonstrated that MTX-induced apoptosis in Hep3B cells occurred through the caspase-9/-3 cascade pathway (Fig. 3A and C). These previous studies indicate that MTX induces different caspase pathways in different hepatoma cell lines. HepG2 is a p53 wild-type hepatoma cell line, while Hep3B is a p53-deficient hepatoma cell line (68,69). Thus, we suggest that p53 may be a possible reason for why the caspase-8/-3 pathway was activated in the MTX-treated HepG2 cells, while the caspase-9/-3 pathway was activated in the MTX-treated Hep3B cells.

Previous studies have demonstrated that MTX-induced cell cytotoxicity is associated with increases in reactive oxygen species (ROS) (35–37). The present study also indicated that MTX-induced H_2_O_2_ level increases may be one factor resulting in cell growth inhibition. On the other hand, vitamin C can reduce oxidative stress against ROS-induced cell damage (43–47). Here, we also demonstrated that vitamin C did reduce MTX-induced increases in H_2_O_2_ levels. However, vitamin C did not inhibit MTX-induced cell cytotoxicity in Hep3B cells. On the contrary, vitamin C assisted low-dose MTX to exhibit a strong cytotoxic effect in Hep3B cells. Similarly, recent studies also indicated that vitamin C can enhance anticancer agents to exert a strong cytotoxic effect on cancer cells, although the mechanisms remain unknown (48,70–72). Thus, MTX-induced increases in H_2_O_2_ levels may be one of the factors resulting in cytotoxicity noted in MTX-treated Hep3B cells. There are various unclear MTX-induced death signals that remain to be studied. Regardless, a combination treatment of vitamin C and low-dose MTX may be a potential method for hepatoma cancer therapy.

Overall, the present study first demonstrated that MTX induces an increase in H_2_O_2_ levels and activates the caspase-9/-3 cascade pathway to cause apoptosis in Hep3B cells. Importantly, a combination treatment of vitamin C and low-dose MTX exerted a strong anticancer effect in Hep3B cells. This treatment method may be useful for future clinical cancer therapy.
